# Improving resident physicians' knowledge of the ABCDEF bundle through a multimodal teaching intervention

**DOI:** 10.5116/ijme.68a2.e3a2

**Published:** 2025-08-29

**Authors:** Priscilla Garcia, Katherine M. Quiñones Cruz, Taylor Ramsaroop, Israel Acosta Sánchez, Christian Ascoli, Tirsa M. Ferrer Marrero

**Affiliations:** 1 Department of Medicine, Division of Pulmonary, Critical Care, Sleep and Allergy, University of Illinois Chicago College of Medicine, Chicago, IL, USA; 2 Section of Nephrology, Department of Medicine, University of Chicago, IL, USA; 3 Department of Medicine, University of Illinois Chicago College of Medicine, Chicago, IL, USA; 4 Department of Medicine, Division of Pulmonary, Critical Care, and Sleep Medicine, Medical College of Georgia, Augusta, GA, USA

**Keywords:** Resident physicians, ABCDEF bundle, multimodal teaching, Intensive Care Unit (ICU), educational intervention, assess prevent and manage pain

## To the Editor

The ABCDEF Bundle (Assess, prevent, and manage pain, Both spontaneous awakening and ventilator breathing trials, Choice of analgesia and sedation, Delirium - assess, prevent, and manage, Early mobility and exercise, Family engagement and empowerment) is an intervention that can be applied to all Intensive Care Unit (ICU) patients.^1^^,^^2^ The Society of Critical Care Medicine (SCCM) recommends its daily implementation to maximize patient outcomes.^1^^,^^3^ It decreases in-hospital mortality, delirium, physical restraint usage, ICU readmissions, and discharges to rehabilitation facilities.^1^^,^^3^

Despite its benefits, adherence rates are suboptimal, with utilization rates reported as low as 67%.^4^ This highlights a gap in patient care that needs to be continuously addressed with interventions to improve adherence. A nationwide survey demonstrated a significant relationship between providers' attitudes and adherence to Bundle implementation. The odds of adherence were lower with perceptions of a high workload burden.^5^ Thus, understanding providers' attitudes can guide interventions to foster Bundle implementation. ^5^^,^^6^

Resident physicians are key members of the ICU team, making their knowledge and attitude toward the Bundle crucial areas of study. In the ICU, we rely on nurses to implement the Bundle following protocols written by the ICU multidisciplinary committees. Our residents run a checklist for every patient, which includes elements of the Bundle, and discuss the details with the nurses. However, the pre-survey underscored a knowledge gap among residents, prompting a teaching intervention to improve their Bundle knowledge, as measured in a post-survey. We report on our experience implementing the teaching intervention and provide details about the trainees' overall knowledge before and after the intervention.

This is an IRB-exempt pre- and post-intervention project conducted at the University of Illinois Hospital, an urban tertiary referral and academic university-based hospital in Chicago, Illinois, USA. The intervention was a multimodal teaching program to impact residents' knowledge about the Bundle and their ability to identify implementation barriers. The intervention included 1) a hybrid conference, 2) ICU round discussions in collaboration with attendings, and 3) dissemination of the SCCM ICU Liberation Poster.^7^

Every resident rotating through an 18-bed medical ICU received an electronic link to an anonymous, voluntary ten-question pre-survey. After the intervention, residents received an electronic link to an anonymous, voluntary seventeen-question post-survey. Both surveys collected demographic information, asked questions about the Bundle and its components, inquired about perceptions of Bundle implementation, assessed prior knowledge acquisition, and queried preferred learning methods. The post-survey evaluated the teaching intervention. The data was analyzed with descriptive statistics and percentages to express the distribution frequency.

Forty-three (20%) of 217 trainees answered the pre-survey and 86% were in the categorical Internal Medicine (IM) residency program. Twelve (28%) were PGY1, seventeen (40%) PGY2, eleven (26%) PGY3, and three (7%) PGY4. The respondents' top future areas of interest were hospitalist medicine and cardiology. Most (91%) answered they were unsure what the Bundle was, including senior residents and those interested in a pulmonary and critical care medicine (PCCM) fellowship. Only 7% correctly identified the Bundle's elements, and 14% correctly identified the elements not belonging to it.

Regarding pre-survey perception of Bundle implementation, 35 (81%) residents did not know what the Bundle was or did not answer. Two (5%) believed it was implemented, and 6 (14%) responded otherwise. Among those who knew about the Bundle, lack of protocol awareness (17%) and knowledge of who is responsible for its execution (12%) were identified as top barriers to implementation. Among all pre-survey respondents, the preferred ways of learning about the Bundle were during ICU rounds (40%), conferences (26%), and from co-residents/fellows or self-learning (19%).

Thirty (14%) of 217 trainees answered the post-survey, and 87% were in the categorical IM residency program. Thirteen (44%) were PGY1, four (13%) PGY2, nine (30%) PGY3, three (10%) PGY4, and one (3%) PGY5. The respondents' top future areas of interest were cardiology, gastroenterology, and hospitalist medicine. In the post-survey, most 22 (73%) knew what the Bundle was, with only 27% answering they were unsure. Nine (30%) learned about it at the noon conference, three (10%) from co-residents/fellows, nine (30%) on ICU rounds, and one (3%) through self-learning.  Most 21 (70%) correctly identified the Bundle elements, and 23 (77%) correctly identified the element not belonging to it. There were no apparent differences in the answers from senior residents and those interested in PCCM. In terms of post-survey Bundle implementation, 9 (30%) residents did not know what the Bundle was or did not answer. Fifteen (50%) believed it was implemented, and 6 (20%) otherwise.

The top identified barriers included a lack of protocol awareness 10 (19%), a lack of communication and coordination 8 (15%), and an increase in workload 6 (11%). Eleven (37%) identified element "E" as the element with more implementation barriers, followed by seven for the element "F" (23%). Eighteen (60%) agreed or strongly agreed that the teaching interventions were effective, while nine (30%) remained neutral.

Statistical analysis was performed using GraphPad Prism 10.4.2 software. Questions assessing Bundle knowledge showed significant improvement in the respondents' knowledge in the post-survey as compared to the pre-survey. Identification of the element "C" showed improvement (χ²(3, N=73) = 35.19, p < 0.0001). Identification of the element not belonging in the Bundle showed improvement (χ²(1, N=73) = 29.03, p < 0.0001). Additionally, perception of ICU implementation increased significantly in the post-survey (χ²(1, N=73) = 20.78, p <0.001). For this question, there was one non-respondent in both the pre- and post-survey.

The pre-survey underscored the need for an educational intervention. This is noteworthy as a lack of knowledge is an obstacle to the effective implementation of the Bundle.^8^ Carrothers and colleagues surveyed ICU staff, and most reported barriers were related to resources and coordination, but 11% identified knowledge deficits as a barrier.^9^ Educating residents on the Bundle could increase their involvement in its implementation, potentially increasing overall adherence rates.

In line with our hypothesis, following the teaching intervention, the post-survey showed a statistically significant improvement in knowledge as respondents could identify the Bundle's elements.

Our study builds upon previous research exploring residents' educational needs. The pre-survey served as a needs assessment to inform the intervention's structure by evaluating current knowledge and preferred learning methods. The post-survey fostered reflective practice since most participants could recognize whether the Bundle had been implemented and the barriers, which were similar to those barriers published by other authors.^4^^, ^^10^^, ^^11^

Pinto and colleagues showed the benefits of promoting learning about the Bundle since, after providing an explanatory document, most surveyed nurses agreed on its clinical benefits, highlighting the importance of an educational intervention and cultural changes towards the Bundle.^12^

The exploration of baseline knowledge and learning needs has been performed before. However, our project is unique because it focuses on a population that, to our knowledge, is often underrepresented in studies assessing providers' perspectives toward the Bundle. Remarkably, the intervention was guided by the pre-survey and residents' preferred learning methods, which explains its high acceptance.

Although our residents run a checklist for each patient, the pre-survey revealed a knowledge deficit and their inability to identify Bundle implementation or barriers encountered. It is possible that they identified each element as a separate intervention but failed to recognize them as part of an evidence-based Bundle. This observation could be due to varying ICU exposure and attending practice styles. The post-survey showed a significant increase in knowledge and recognition of the Bundle.

Notably, our intervention only affected some residents, and whether those who answered the surveys also participated in the interventions is unknown. Since we cannot match the surveys with participants, we cannot explore the differences between those who answered both surveys and the differences between the surveys of those who participated in the interventions. However, we conjecture that our results are from those who would have answered both surveys and participated in the interventions. It is also plausible that those who answered the surveys were interested in this topic and likely to answer correctly, reflecting not on the intervention's impact but the knowledge acquired a priori.

The low participation rate could introduce a selection bias, a limitation inherent in the methodology, given that participation was voluntary and anonymous to protect residents' identities. Another limitation is that this is a single-site study due to time constraints and lack of funding, which restricts the generalizability of our results.

Another limitation is the failure to assess the need for a longitudinal educational intervention to address challenging barriers. Most respondents identified element "E" as the most difficult to implement, which is consistent with other studies. ^5^^,^^13^ Element "F" was considered the second most challenging. This finding could be influenced by the low participation rate or considered challenging due to residents' demanding schedules. Our residents actively communicate with families, but shared decision-making is a complex competency.^14^ However, the gap between theoretical learning and practice can be closed through collaborations with the Palliative Medicine team and simulation-based educational activities.

As a one-time intervention might be insufficient, we must consider sustained and comprehensive educational strategies. This study can inform future research on evaluating knowledge retention and the effectiveness of longitudinal interventions.

Overall, the limitations discussed can be addressed through future multicenter randomized studies to ensure the accuracy and reliability of the results, including trainees rotating through different types of ICUs in academic institutions.

In summary, the pre-survey underscored the need for an educational intervention to improve residents' knowledge about the ABCDEF Bundle. The post-survey showed that most respondents knew about the Bundle and could identify the barriers to its implementation. Although we cannot establish an association given the methodology, we could still speculate that our multimodal teaching intervention seems effective.

### Conflicts of Interest

The authors declare that they have no conflict of interest.
